# Divergence in expression of a singing-related neuroplasticity gene in the brains of 2 *Ficedula* flycatchers and their hybrids

**DOI:** 10.1093/g3journal/jkae293

**Published:** 2024-12-13

**Authors:** David Wheatcroft, Niclas Backström, Ludovic Dutoit, S Eryn McFarlane, Carina F Mugal, Mi Wang, Murielle Ålund, Hans Ellegren, Anna Qvarnström

**Affiliations:** Department of Ecology and Genetics, Animal Ecology, Uppsala University, 752 36 Uppsala, Sweden; Department of Ecology and Genetics, Evolutionary Biology, Uppsala University, 752 36 Uppsala, Sweden; Department of Zoology, Stockholm University, 619 95 Stockholm, Sweden; Department of Ecology and Genetics, Evolutionary Biology, Uppsala University, 752 36 Uppsala, Sweden; Department of Ecology and Genetics, Evolutionary Biology, Uppsala University, 752 36 Uppsala, Sweden; Department of Zoology, University of Otago, Dunedin 9016, New Zealand; Department of Ecology and Genetics, Animal Ecology, Uppsala University, 752 36 Uppsala, Sweden; Department of Biology, York University, M3J 1P3 Toronto, Canada; Department of Ecology and Genetics, Evolutionary Biology, Uppsala University, 752 36 Uppsala, Sweden; Laboratory of Biometry and Evolutionary Biology (LBBE), CNRS, UMR 5558, University of Lyon 1, Villeurbanne 69622, France; Department of Ecology and Genetics, Evolutionary Biology, Uppsala University, 752 36 Uppsala, Sweden; Department of Ecology and Genetics, Animal Ecology, Uppsala University, 752 36 Uppsala, Sweden; Department of Ecology and Genetics, Evolutionary Biology, Uppsala University, 752 36 Uppsala, Sweden; Department of Ecology and Genetics, Animal Ecology, Uppsala University, 752 36 Uppsala, Sweden

**Keywords:** *Ficedula*, gene expression, song learning, speciation, *STXBP4*, *SYT4*

## Abstract

Species-specific sexual traits facilitate species-assortative mating by reducing mating across species and reducing hybrid sexual attractiveness. For learned sexual traits, such as song in oscine birds, species distinctiveness can be eroded when species co-occur. Transcriptional regulatory divergence in brain regions involved in sensory learning is hypothesized to maintain species distinctiveness, but relatively few studies have compared gene expression in relevant brain regions between closely related species. Species differences in song are an important premating reproductive barrier between the collared (*Ficedula albicollis*) and pied flycatcher (*F. hypoleuca*). Here, we compare brain gene expression in adult males from each species and their naturally occurring F1 hybrids. We report overall conserved expression across species in a portion of the brain containing regions and nuclei known to be involved in song responses and learning. Further, among those genes that were differentially expressed between species, we find largely intermediate expression in hybrids. A single gene, *SYT4* (synaptotagmin 4), known to be singing-associated, both was differentially expressed and has a putative upstream transcriptional regulatory factor containing fixed differences between the 2 species. Although a finer-scale investigation limited to song-specific regions may reveal further species differences, our findings provide insight into regulatory divergence in the brain between closely related species.

## Introduction

Differences in sexual signals play a key role in maintaining reproductive barriers between species by allowing individuals to discriminate against potential heterospecific mates (Coyne and Orr 2004). Revealing the mechanisms that promote and maintain differences in sexual signals across species is therefore critical to our general understanding of how species in such groups are formed and maintained. As mating traits diverge relatively quickly between closely related species, such differences are commonly thought to be underlain by changes in regulatory sequences, rather than coding sequences (Romero *et al*. 2012). This is particularly likely for learned mating traits, such as the song produced by oscine birds, since the coding sequences of brain-expressed genes are often highly conserved (Hadley *et al*. 2006; Dumas *et al*. 2021). Songs differ significantly, even between closely related species (Price 2008), but relatively few studies have characterized species differences in gene expression in brain regions involved in auditory perception and learning (Drnevich *et al*. 2012; Balakrishnan *et al*. 2014; Suzuki *et al*. 2014; Wang *et al*. 2019). Here, we use RNA sequencing to compare gene expression in the brain between 2 closely related bird species and their naturally occurring hybrids.

Song learning in birds begins with a sensory learning phase during which juveniles listen to and memorize songs to which they are exposed (Bolhuis and Moorman 2015). Sensory learning depends not only on an individual’s specific song experience (e.g. Grant and Grant 1996), but also any predispositions or biases that influence the types of songs are likely to be particularly stimulating (e.g. Nelson and Marler 1993; Wheatcroft and Qvarnström 2015). For example, juvenile birds of some species are particularly responsive to the songs of their own species (Nelson and Marler 1993; Shizuka 2014) or, even, population (Nelson 2000; Hudson *et al*. 2019; Wheatcroft *et al*. 2022). In the following sensory-motor learning phase, singers refine the songs they produce through comparison with what they have previously learned until they develop adult song (Bolhuis and Moorman 2015). In some species, called “open-ended learners,” these phases continue into adulthood, meaning that individuals can incorporate new elements into their songs based on ongoing experience (Brenowitz and Beecher 2005). For such species, predispositions to learn specific song elements may be important for maintaining species distinctiveness throughout life.

**Fig. 1.**
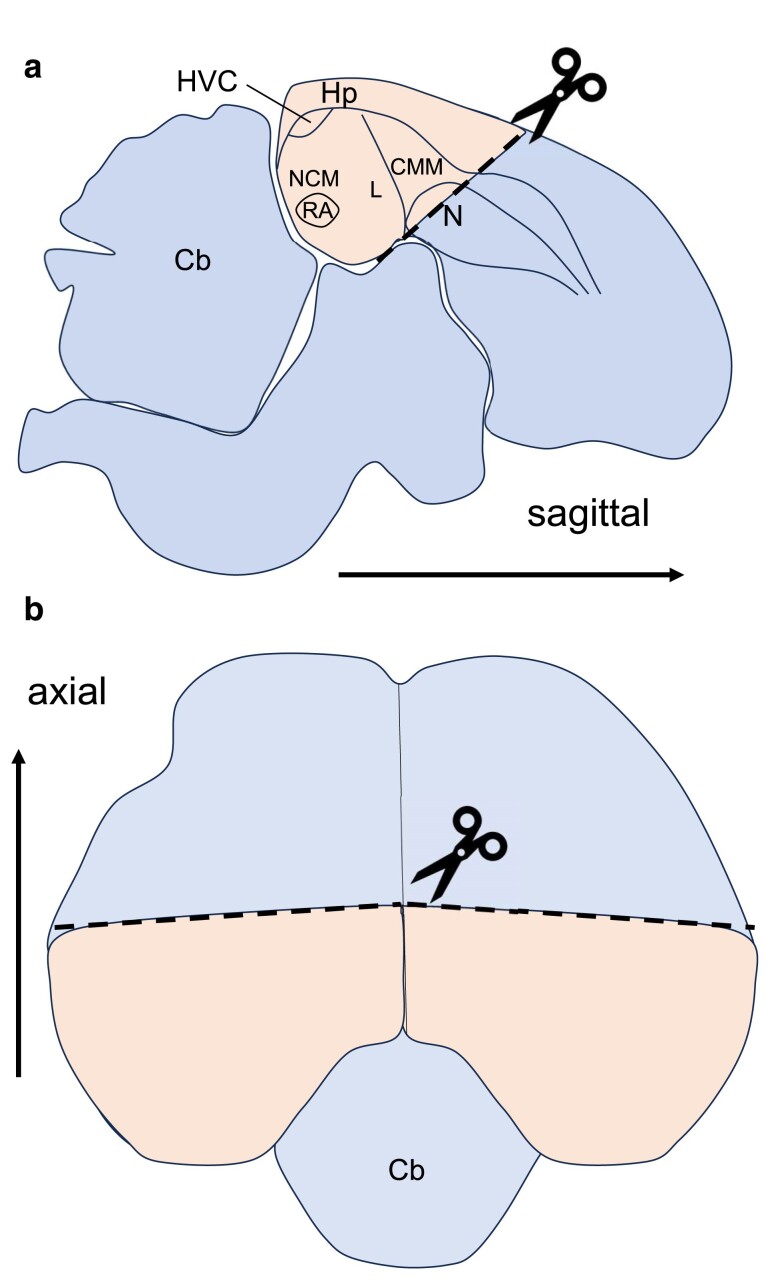
Schematic a) sagittal cross-section and b) axial view of a songbird brain, showing the dissected portion of the caudal telencephalon. The thick dashed line denotes the rough dissection markers used at midline. Cb = cerebellum, Hp = hippocampus, HVC (proper name), NCM = caudomedial nidopallium, L = Field L, CMM = caudal medial mesopallium, RA = robust nucleus of the arcopallium (Reiner *et al*. 2004).

Research over the last decades has suggested that song differences between closely related species are maintained in part by sensory-perceptual differences between species that make individuals more likely to perceive, learn, and produce conspecific song elements (Marler 1997; Wright *et al*. 2004; Mundinger and Lahti 2014; Suzuki *et al*. 2014; Wheatcroft and Qvarnström 2015). First, the auditory lobule, the avian equivalent to the mammalian auditory cortex, develops heightened responsiveness to conspecific sounds early in development and is thought to underlie species-specific perception of sounds (region containing NCM, CMM, and L in Fig. 1; Wheatcroft and Qvarnström 2015). Second, species differences in the morphology of and gene regulation in brain regions involved in the formation and recall of song memories, including the song control system (Fig. 1), are thought to influence the accuracy and flexibility of song learning (Devoogd *et al*. 1993; Szekely *et al*. 1996; Airey *et al*. 2000; Bailey *et al*. 2002; Suzuki *et al*. 2014). A comparison between zebra finches (*Taeniopygia guttata*), double-barred finches (*T. bichenovii*, estimated divergence time, 5–9 my; Olsson and Alström 2020), and their captive-bred hybrids concluded that interspecific divergence in song learning is in part explained by divergence in transcriptional regulation of neuroplasticity genes in song control nuclei (Wang *et al*. 2019). As a result, divergence in gene expression in auditory and song control brain regions may be an important driver of species differences in song learning and production.

The collared (*Ficedula albicollis*) and pied flycatcher (*F. hypoleuca*) are 2 closely related (<1 mya; Nadachowska-Bryzska *et al*. 2013) songbird species. The 2 species co-occur on the Baltic island of Öland, and while mating is mostly species-assortative, ∼6% of breeding pairs are mixed (i.e. 1 adult from each species; Qvarnström *et al*. 2010). Song differences between the species play an important role in species recognition and mate choice (Wheatcroft and Qvarnström 2017a, 2017b), and individuals producing intermediate songs suffer an increased risk of mixed-species pairing (Qvarnström *et al*. 2006). These cross-species pairings involve substantial fitness costs to both partners: F1 hybrid females are sterile (Svedin *et al*. 2008) and hybrid males experience highly reduced, if any, fertility (Ålund *et al*. 2013). As a result, understanding the mechanisms driving song differences between the species will provide insight into the maintenance of premating reproductive barriers between the flycatcher species. Two sources of evidence suggest that song differences between species are underlain in part by interspecific genetic variation. First, flycatcher nestlings that have been incubated and raised by heterospecific parents discriminate in favor of conspecific songs (Wheatcroft and Qvarnström 2017a) and produce largely conspecific songs as adults (Eriksen *et al*. 2009). Second, hybrid flycatchers produce songs that are largely intermediate between those of either parental species (Gelter 1987), despite generally having collared flycatcher social fathers (Vallin *et al*. 2012). As argued above, a genetic basis for song differences between species is likely to be driven by regulatory divergence in auditory and song control brain regions. However, we lack an understanding of how gene expression in relevant brain regions diverges between closely related species in the wild, such as the pied and collared flycatcher. Although song learning is typically thought to primarily occur in juvenile birds, both flycatcher species are so-called open-ended song learners, meaning that they incorporate new elements into their song repertoires as adults (Eriksen *et al*. 2011; Vaskuti *et al*. 2022). As a result, species-specific patterns of gene expression in adult birds may influence song phenotypes.

Here, we first characterize gene expression in the caudal telencephalon. This brain portion contains regions (auditory lobule) and nuclei (HVC, RA) known to be involved in auditory responses and song learning/production, respectively, in adult males as well as a variety of regions whose functions are not related to song (Fig. 1). Next, we compare expression in this brain sample among wild adult males from each species and naturally occurring F1 hybrids. Finally, we use genome resequencing data to reveal fixed differences in *cis*- and *trans*-acting regulatory factors of these differentially expressed genes.

## Materials and methods

### Study individuals

We captured 14 male *Ficedula* flycatchers from a nestbox population on the Baltic island of Öland (57°10′ N, 16°56′ E) during the courtship phase of the breeding season in May 2014. Species identity was assessed using characteristic differences across species in songs, alarm calls, and plumage (Qvarnström *et al*. 2010). Putative hybrids were determined by their abnormal or intermediate plumage. Species and hybrid identity was later confirmed after RNA sequencing by comparing SNPs with known fixed differences between collared and pied flycatchers (Mugal *et al*. 2020). One of the putative hybrid males was determined to be a collared flycatcher and was subsequently excluded from the analysis. In total, we caught and identified 5 pied and 5 collared flycatcher males and 3 hybrid males (all 3 with collared flycatcher fathers and pied flycatcher mothers; Mugal *et al*. 2020). Most individuals (*N* = 8 out of 13) had rings from previous years and were confirmed to be at least 2 years old, while 2 pied and 2 collared flycatcher males and 1 hybrid male were of unknown age.

All males were kept in outdoor, covered 3 × 3 × 2 m aviaries. The males were kept as part of a larger experiment evaluating testes gene expression: all 5 pied flycatcher males and 3 of the collared flycatcher males were paired with conspecific females, while hybrid males were paired with collared flycatcher females and 2 collared flycatcher males were unpaired. Each aviary was provided with a nest box, nest building material, and ad libitum mealworms and water. Birds were kept in these conditions for at least 1 week prior to tissue collection to minimize potential variation in gene expression.

### Tissue collection

All sacrifices and dissections occurred on the same morning. The evening before, the birds were placed into dark, quiet, climate-controlled conditions (28°C), where they were kept for 8 h. In the morning, they were placed in dark, quiet cages. No male sang during this period, but, as part of another experiment, each male was exposed to a 1-min conspecific song playback (hybrids received either pied or collared flycatcher playback) before euthanizing them by decapitation. We note that song-induced expression of even immediate early genes requires up to 30 min of continuous song playback, so this playback could not influence our results (Wada *et al*. 2006). Brains were rapidly removed from skulls, and dissections were performed using light microscopy over an ice pack to keep the tissue as cold as possible. Dissections were alternately performed by D.W. and N.B., ensuring a balance across species. We first bisected each brain down the midline, and then, for each hemisphere, we used the delineation visible at midline between the pallium and subpallium together with the orientation of the thalamus to dissect the entire caudal dorsal portion of the telencephalon (Fig. 1). The caudal telencephalon contains the entire auditory lobule (Cheng and Clayton 2004), hippocampus, song control system nuclei RA and HVC, as well as medial and lateral parts of the caudal telencephalon, including the arcopallium, mesopallium, nidopallium, and parahippocampus, while avoiding adjacent thalamic and subpallial regions. We note that these adjacent regions typically highly express tyrosine hydroxylase (*TH*), the rate-limiting enzyme for dopamine synthesis, while *TH* was lowly expressed in our samples (*N* = 13, 2.14 ± 3.09 mean transcripts per million reads ± SD). After each dissection, which took no longer than 20 min per individual, samples from both hemispheres were placed into a single Eppendorf containing RNA*later*, flash-frozen in liquid nitrogen, and subsequently stored at −80°C until RNA extraction. In addition to the brain samples, we dissected the heart, liver, kidney, and testis from each individual and stored them similarly. These experimental procedures were approved by the Swedish Board of Agriculture (Jordbruks Verket—Linköping Djurförsöksetiska DNR 21-11).

### RNA sequencing and expression analyses

RNA extractions, library preparation, and sequencing are outlined in Mugal *et al*. (2020). Briefly, tissues were homogenized, and total RNA was extracted using Qiagen RNeasy kits (Qiagen). RINs for all samples were above 8.8. RNA-seq libraries were prepared using TruSeq stranded mRNA kits (Illumina, RS-122-2103), and paired-end 125-bp reads were sequenced on an Illumina HiSeq instrument (34–47 million reads per sample). In addition, the methods used to map reads to the flycatcher genome, count reads, and perform differential expression analysis are outlined in Mugal *et al*. (2020). Reads were mapped onto the FicAlb 1.5 collared flycatcher assembly (GenBank accession: GCA_000247815.2) using STAR v.2.5.1b (Dobin *et al*. 2013). Differential expression analysis was performed using DESeq2 in R (Love *et al*. 2014). After differential expression analysis, we considered a gene to have a caudal telencephalon differential expression if the gene was differentially expressed between the species in the caudal telencephalon, but either not differentially expressed in any of the other 4 tissues or, if the gene was differentially expressed in another tissue, the change in the caudal telencephalon was of opposite direction (i.e. collared flycatcher > pied flycatcher in the caudal telencephalon, and pied flycatcher > collared flycatcher in the other tissue).

### Tissue enrichment

All analyses were performed in R v4.3 (R Core Team 2023). To distinguish true gene expression from noise, we transformed transcript-per-million reads using a $\log_{2}\;(\mathrm{tpm}+1)\;$ transformation. We considered a gene to be expressed in 1 of the 5 sequenced tissues (caudal telencephalon, heart, liver, kidneys, and testis) if the average number of transformed transcripts-per-million reads was at least 1 for that tissue. For each gene expressed in the caudal telencephalon, we obtained a measure of caudal telencephalon-specific expression by calculating the fold increase in expression of each gene in the caudal telencephalon relative to the other tissue in which it was most highly expressed. Using 5× and 50× cutoffs, we generated subsets of genes enriched and highly enriched in the caudal telencephalon, respectively.

### Gene ontology analyses

We assigned functional categories to gene sets using human-derived gene ontology (GO) terms. We limited our analysis to flycatcher genes having one-to-one human orthologs and acquired UniProt protein ids for each gene using getBM in biomaRt v 2.48.0 (Durinck *et al*. 2009). Then, for each UniProt id, we assigned UniProt-SwissProt manual GO annotations derived for humans (The UniProt Consortium 2017). We determined functional associations of the set of genes that were enriched and highly enriched in the caudal telencephalon, grouping all individuals together, using runGSAhyper implemented in the R package piano v 2.18.0 (Väremo *et al*. 2013). For each GO category, a *P*-value is assigned using Fisher’s test comparing the number of genes in the set that are present/absent in the category with the number of genes not in the set that are present/absent in the category. *P*-values for associations were adjusted for multiple comparisons using the false discovery rate correction (Benjimani and Hochberg 1995). We determined differentiation of functional associations between species using runGSA in piano. For each GO category, a *P*-value is determined using resampling based on the adjusted *P*-value and direction of differential expression. We conducted identical tests on the following subsets of genes: (1) all genes expressed in the caudal telencephalon, (2) genes enriched in the caudal telencephalon, and (3) genes highly enriched in the caudal telencephalon.

### Weighted gene co-expression network analysis

Weighted gene co-expression network analysis (WGCNA) is used to identify sets of genes sharing a similar expression pattern, termed “modules.” We performed WGCNA using our expression data set in the caudal telencephalon to identify modules associated with species identity. Background details on the methodology can be found in Zhang and Horvath (2005). All analyses were performed in R using WGCNA v.1.72 (Langfelder and Horvath 2008).

**Fig. 2.**
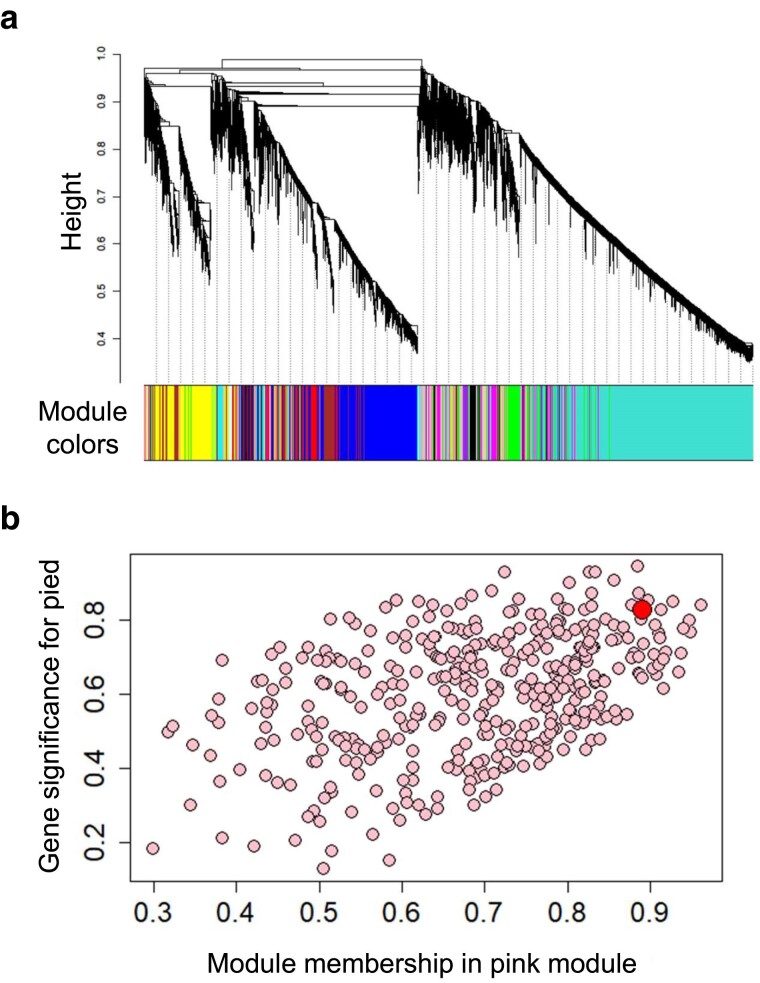
a) Dendrogram of 11,845 brain-expressed genes and their module assignments from WGCNA. b) Genes in the pink module. Scatterplot of gene significance (i.e. association between expression of each gene with species) against its module membership (i.e. correlation between expression of each gene with the module eigengenes) for all 403 genes included in the pink module. The pink module was determined through WGCNA to be significantly associated with species. Placement of *SYT4* given by red-filled circle.

We removed lowly expressed mRNAs by filtering out those with mean log(tpm + 1) values lower than 1 across all 10 pure species individuals, leaving a total of 11,845 expressed mRNAs. We constructed a signed-hybrid network using our expression data from the caudal telencephalon from the 10 pure species males using a soft-thresholding power of 4 and a minimum module size of 30. From this network, modules were defined by default cutoffs of branches from the network dendrogram (Fig. 2a). Module eigengenes summarize highly correlated module genes, while module membership quantifies a gene’s association with a given module. Second, we related regulation of genes within modules with species identity. Modules were associated with species identity by obtaining Pearson’s correlation coefficients of module eigengenes from each module with species identity (collared flycatcher = 0, pied flycatcher = 1), and obtaining *P*-values from a Student’s *t*-distribution. Third, we associated species-correlated modules with functional categories similarly to GO enrichment analyses described above, using the runGSAhyper function comparing the presence/absence of genes in the module associated with human-derived GO terms.

### Molecular determinants of RIMS3 and SYT4 expression divergence

Following Mugal *et al*. (2020), we used the R package RPASE (Wang *et al*. 2018) to determine overall patterns of allele-specific expression (ASE) in the caudal telencephalon and, thereby, determine whether species divergence in expression is driven by divergence in *cis*- vs *trans*-regulation. The drivers of expression differences could differ across brain regions (e.g. Wang *et al*. 2019) or even cell types (e.g. van der Wijst *et al*. 2018). As a result, conclusions about ASE based on bulk RNA-seq data from a large portion of the brain may not apply to smaller regions and nuclei contained in this larger portion. However, we here use this analysis to guide a search for potential regulatory drivers of species differences, which enabled us to identify putative *trans*-acting factors. In addition, in order to account for the uncertainty in applying ASE from bulk RNA-seq to smaller regions, we used polymorphism data from Burri *et al*. (2015) to identify potential conserved regulatory regions in the proximity of both *SYT4* and *RIMS3*.

For *SYT4* and *RIMS3*, we identified fixed differences between the species in putative *trans*-acting factors. We did this by generating a list of putative upstream regulators of each gene and upstream regulators of those regulators generated through ingenuity pathway analysis (QIAGEN Inc., www.qiagenbio-informatics.com/products/ingenuity-pathway-analysis; Krämer *et al*. 2014). In total, we evaluated 163 and 104 putative upstream regulators of *SYT4* and *RIMS3*, respectively. To calculate fixed differences in target genes, polymorphism data for 19 collared and 19 pied flycatchers from Öland were retrieved from Burri *et al*. (2015). All polymorphisms that were not covered by at least 1 read over all individuals were removed. Fixed differences between the 2 species in the gene body and ±5-kb windows were then extracted using in-house scripts. For each gene with fixed, nonsynonymous differences, we located the position of the amino acid substitution in the protein sequence and compared this with annotated functional protein domains in the SMART (Letunic and Bork 2017) and InterPro (Mitchell *et al*. 2019) protein databases. Concurrently, we used multiple online tools (MARCOIL, Delorenzi and Speed 2002; Paircoil2, McDonnell *et al*. 2006; Multicoil2, Trigg *et al*. 2011; PCOILS, Gruber *et al*. 2006; DeepCoil, Ludwiczak *et al*. 2019; Zimmermann *et al*. 2018) to predict the precise location of the amino acid substitution within functional domains of the proteins.

Finally, we conducted multiple alignments of STXBP4 protein sequences using the blastp suite (blast.ncbi.nlm.nih.gov). We queried the amino acid sequence until the first stop codon and evaluated variation at the variable amino acid site using COBALT (blast.ncbi.nlm.nih.gov accessed June 2022; Altschul *et al*. 1997). We extracted the top 250 hits in passerine birds and identified 152 species with amino acid sequences. We constructed a maximum clade credibility tree for all 152 species using TreeAnnotator based on sampling 1,000 phylogenetic trees with the Hackett backbone from birdtree.org (Jetz *et al*. 2012). A maximum clade credibility tree was constructed in TreeAnnotator v1.10 (Suchard *et al*. 2018). We determined the ancestral character state at the variable amino acid site through parsimony (*ancestral.pars*) and maximum likelihood (*ancestral.pml*) in phangorn 2.11 (Schliep 2011).

## Results

### Characterization of gene expression in the caudal telencephalon

We compared expression in the caudal telencephalon (Fig. 1) with 4 other tissues (heart, kidney, liver, and testis) of collared, pied, and hybrid flycatcher males to determine genes that showed enriched expression in the caudal telencephalon relative to other tissues. A total of 13,581 genes were expressed in at least 1 of the 5 tissues (Supplementary Table 1). Of these, the majority of genes (88%, *N* = 11,845) were also expressed in the caudal telencephalon and a minority (4%, *N* = 569 genes) were expressed only in the caudal telencephalon. Expression of 1,860 genes was enriched (i.e. 5× fold-change) in the caudal telencephalon relative to other tissues, and 654 were highly enriched (i.e. 50× fold-change; Supplementary Table 1). Genes that were enriched and highly enriched in the caudal telencephalon were associated with GO terms relevant to the nervous system including chemical synaptic transmission, calcium ion-regulated exocytosis of neurotransmitter, nervous system development, neuropeptide signaling pathway, and regulation of synaptic plasticity (a complete list of significant GO terms in Supplementary Table 2).

### Conserved gene expression between species

Gene expression in the caudal telencephalon was highly similar between species. Only 52 genes (out of 11,845 expressed in the caudal telencephalon) were differentially expressed between the 2 species (Supplementary Table 3). Of these, 27 showed a pattern of caudal telencephalon-specific differential expression, meaning they were not differentially expressed in the other, nonbrain tissues investigated here (Table 1; Supplementary Table 3). Half of these genes (*N* = 14) had higher expression levels in collared flycatchers. Only 2 of the genes showing caudal telencephalon-specific differential expression were also highly enriched in the caudal telencephalon relative to all other tissues, *RIMS3* (regulating synaptic membrane exocytosis 3, fold increase in the caudal telencephalon compared with other tissues: 163×) and *SYT4* (synaptotagmin 4, fold increase in the caudal telencephalon: 590×; Supplementary Table 1). The majority of the variance in expression of both *RIMS3* (58%; SSspecies/SStotal from ANOVA) and *SYT4* (68%; SSspecies/SStotal from ANOVA) was explained by variation between, rather than within species.

**Table 1.** Genes showing differential expression in the caudal telencephalon between collared and pied flycatchers.

Gene id	Fold increase in brain^b^	Mean tpm ± SD in CF and PF	DE pattern	*P*-value^c^	Pattern in hybrids
*SYT4*	590	93.55 ± 22.80; 158.99 ± 27.20	P > C	<0.001	P > H > C
*RIMS3*	163	23.76 ± 4.28; 14.26 ± 4.71	C > P	0.019	C > H > P
*COL24A1*	2	5.22 ± 0.68; 6.97 ± 1.00	P > C	0.030	P > H > C
*RWDD3*	2	36.08 ± 1.69; 52.75 ± 5.85	P > C	<0.001	P > H > C
*DDX58*	1	25.70 ± 50.01; 14.63 ± 1.18	C > P	<0.001	C > H > P
*RNF180*	1	22.17 ± 1.46; 18.13 ± 1.54	C > P	0.033	C > H > P
*TERF2*	1	45.74 ± 3.76; 54.60 ± 3.78	P > C	0.036	P > H > C
*TRMT44*	1	65.47 ± 4.46; 43.46 ± 7.63	C > P	0.008	C > H > P
UNK^a^	1	27.59 ± 4.72; 20.34 ± 3.15	C > P	0.046	C > H > P
*WDR77*	1	58.62 ± 3.27; 73.47 ± 3.18	P > C	0.036	P > H > C
*ATG7*	<1	42.08 ± 2.54; 34.03 ± 2.44	C > P	0.013	C > H > P
*CA12*	<1	3.43 ± 1.44; 1.02 ± 0.48	C > P	<0.001	C > H > P
*DEPDC1*	<1	3.65 ± 0.77; 6.42 ± 0.71	P > C	<0.001	P > H > C
*FBP1*	< 1	2.91 ± 0.87; 0.96 ± 0.46	C > P	0.006	C > H > P
*G2E3*	<1	19.95 ± 1.50; 15.52 ± 1.21	C > P	0.013	C > H > P
*GPX8*	<1	5.18 ± 2.22; 0.96 ± 0.31	C > P	<0.001	C > H > P
UNK^a^	<1	2.75 ± 0.56; 6.67 ± 3.39	P > C	<0.001	P > H > C
*MED18*	<1	22.60 ± 3.89; 34.05 ± 6.11	P > C	<0.001	P > H > C
*MISP*	<1	2.10 ± 0.55; 3.54 ± 0.86	P > C	0.012	P > H > C
*MRM2*	<1	15.56 ± 1.39; 6.60 ± 0.90	C > P	<0.001	C > H > P
*OSMR*	<1	0.72 ± 0.22; 1.36 ± 0.49	P > C	0.049	P > H > C
*PEPD*	<1	29.44 ± 3.57; 40.16 ± 4.79	P > C	0.040	P > H > C
*RPP40*	< 1	20.35 ± 4.34; 30.65 ± 3.22	P > C	0.030	P > H > C
*SAMD7*	<1	0.31 ± 0.30; 1.34 ± 0.64	P > C	0.006	P > C > H
*SEMA4D*	<1	40.18 ± 3.35; 31.18 ± 4.62	C > P	0.024	C > H > P
*TRAFD1*	<1	24.16 ± 2.41; 28.49 ± 1.39	P > C	0.044	P > H > C
*ZNF280D*	<1	43.24 ± 4.18; 34.40 ± 1.67	C > P	<0.001	C > H > P

^*a*^Gene was not included in gene set enrichment analysis because it is a novel gene with no human ortholog.

^*b*^Compared with the other tissue in which the gene showed the highest expression.

^*c*^ *P*-values are adjusted for multiple comparisons using false discovery rate.

### Few gene expression differences between species and F1 hybrids

**Fig. 3.**
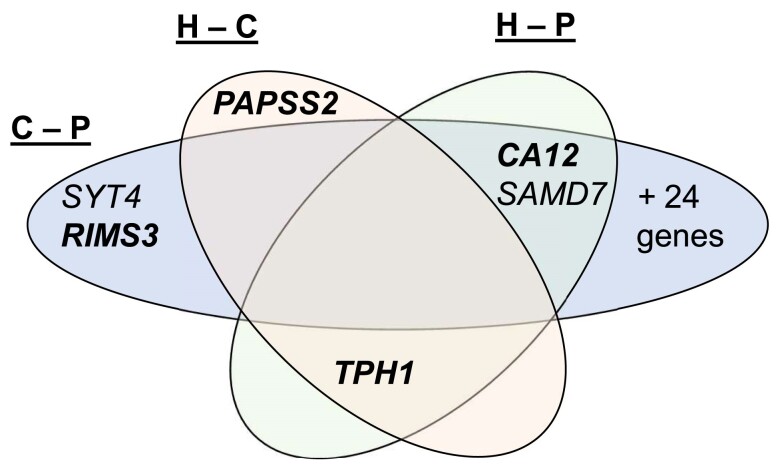
Venn diagram summarizing genes differentially expressed among different contrasts. “C”: collared flycatcher; “P”: pied flycatcher; “H”: F1 hybrids. Bold indicates that gene has greater expression in collared flycatchers. A complete list of genes differentially expressed between collared and pied flycatchers is given in Table 1.

Of the 52 genes that were differentially expressed between collared and pied flycatchers in the caudal telencephalon, all but 1 had expression in F1 hybrids that was intermediate to that of each parental species (Table 2). The single exception to this pattern, *SAMD7*, had lower expression levels than either pure species. Fewer than 10 genes were differentially expressed between hybrids and collared (*N* = 4 genes) or pied flycatchers (*N* = 7), and a single gene, *TPH1*, was differentially expressed between hybrids and both pure species (Table 2; Fig. 3; Supplementary Table 3). Three genes, including *TPH1*, showed a pattern of differential expression specific in the caudal telencephalon relative to the other nonbrain tissues (Table 2), and *TPH1* was the only differentially expressed gene to be enriched in the caudal telencephalon (fold increase: 12×, Supplementary Table 1). For most of the 57 genes that were differentially expressed in the caudal telencephalon either between pure species (52 genes) or hybrids (10 genes, 5 in both comparisons), the variation across hybrid individuals was either less than or similar to the variation across pure species individuals. *TPH1*, noted above, was a clear exception, with 17× the standard deviation across hybrid than pure species individuals. In contrast to *RIMS3* and *SYT4* outlined above, 58% (SSspecies/SStotal from ANOVA) of the variance in expression of *TPH1* was explained by variation across individuals, rather than species.

**Table 2.** Genes showing differential expression in the caudal telencephalon between F1 hybrids and collared and/or pied flycatchers.

Gene id	Fold increase in brain^a^	Mean tpm ± SD	DE pattern	*P*-value^b^	Pattern in pure species
*TPH1*	12	30.57 ± 36.84	H > C; H > P	0.007; 0.031	H > P > C
*CA12*	<1	2.52 ± 0.47	C > P; H > P	<0.001; 0.049	C > H > P
*PAPSS2*	<1	3.31 ± 0.34	H > C	0.005	H > P > C
*SAMD7*	<1	0.08 ± 0.07	P > C; P > H	0.006; 0.003	P > C > H

^*a*^Compared with the other tissue in which the gene showed the highest expression.

^*b*^ *P*-values are adjusted for multiple comparisons using false discovery rate.

### No GO terms significantly associated with expression differences between pure species or their F1 hybrids

We used gene set enrichment analyses to determine whether expression differences in the caudal telencephalon between species were associated with specific functional categories. We found no evidence that any GO terms were differentially enriched across species based either on all brain portion–expressed genes, on genes that were enriched, or highly enriched in the caudal telencephalon relative to other tissues (*P* > 0.3). Likewise, no GO terms were differentially enriched across either species and the F1 hybrids based either on all expressed genes, on genes that were enriched, or highly enriched in the caudal telencephalon relative to other tissues (*P* > 0.4).

### Weighted gene co-expression network analysis

The above analysis relies on gene functional annotations to relate expression patterns with functional changes. An alternative approach, WGCNA, defines clusters or modules of genes whose expression is correlated irrespective of their putative functional associations. We constructed a gene co-expression network using the expression data from the 10 pure species males (Fig. 2a). This analysis identified 16 clusters of co-expressed genes or modules in the caudal telencephalon (Supplementary Table 4). The pink module (403 genes) had the strongest association with species (Supplementary Table 4). In addition, the purple (353 genes) and light cyan modules (221 genes) were also differentially regulated between species, while the other 13 modules had no significant species association (Supplementary Table 4).

*SYT4* was assigned to the pink module (module membership = 0.89, *P* < 0.001) and was among the top 5% of genes in this module for module membership (connectivity with other genes in the module) and the top 7% for gene significance (association with species; Supplementary Table 5; Fig. 2b). *RIMS3* was assigned to the purple module (module membership = 0.76, *P* = 0.011). However, *RIMS3* was outside of the top 40% of genes in the purple module for module membership. We found no significant association between the pink, purple, or tan modules and any GO category (*P* > 0.29). In subsequent analyses, we focus on these 2 genes due to the fact that 2 independent analyses identified them as having species-specific expression as well as the fact that they were highly enriched in the caudal telencephalon relative to other tissues.

### Mechanisms driving divergence in expression in RIMS3 and SYT4

We identified the putative regulatory mechanisms (*cis*- vs *trans*-regulatory) of differences in expression in *RIMS3* and *SYT4* between species by determining ASE in F1 hybrids (Mugal *et al*. 2020). *RIMS3* lacked a sufficient number of phased SNPs to determine whether hybrids display ASE (Mugal *et al*. 2020). As a result, we cannot investigate whether expression divergence within species is due to *cis*- or *trans*-regulatory divergence. However, based on the whole-genome re-sequencing data from Burri *et al*. (2015), we observed no fixed differences between pied and collared flycatchers either in the gene body, 5′ and 3′ UTRs, or in the surrounding 5-kb regions of *RIMS3*, suggesting no clear role of *cis*-regulatory divergence between species (Supplementary Table 3). We therefore explored the potential for species divergence in *trans*-regulatory factors to drive the expression difference between species, generating a list of putative upstream regulators of *RIMS3* as well as putative upstream regulators of those genes, together analyzing 104 genes (Supplementary Table 6). One of these genes, *CREB3L2*, which regulates *CREB3* expression, was determined to have a single nonsynonymous fixed difference between species (Supplementary Table 6). However, this fixed difference was not associated with any of the known functional domains of the CREB3L2 protein (Supplementary Table 7).

Hybrids display no ASE of *SYT4*, consistent with the hypothesis that divergence in *trans*-acting factors underlies species divergence in expression of this gene. As above, based on the whole-genome re-sequencing data from Burri *et al*. (2015), we observed no fixed differences between pied and collared flycatchers either in the gene body, 5′ and 3′ UTRs, or in the surrounding 5-kb regions of *RIMS3*, suggesting against a clear role of *cis*-regulatory divergence between species. We then conducted a similar analysis as above to determine the putative upstream regulators of *SYT4* and upstream regulators of those genes, together analyzing 163 genes (Supplementary Table 6). Four of these genes were identified to have fixed nonsynonymous differences between the species: *NBR1*, *PRKN*, *SNCAIP* (regulates *PRKN*), and *STXBP4* (regulates *STX4*). For *NBR1* (Supplementary Table 7), *PRKN* (Supplementary Table 7), and *SNCAIP* (Supplementary Table 7), the fixed difference was not associated with any known functional domain of the protein. However, for *STXBP4*, the fixed difference results in an amino acid substitution (serine in collared flycatcher to threonine in pied flycatcher) associated with a coiled coil motif (Supplementary Table 7). The location of the substitution is predicted to be either within the coiled coil motif (database: SMART; prediction tools: MARCOIL, Paircoil2, Multicoil2, PCOILS) or within 10 amino acids of the 1st position (database: InterPro; prediction tool: DeepCoil).

Finally, we identified annotated copies of *STXBP4* in the genomes of 152 passerine species (Supplementary Table 8). The majority of these species (*N* = 140) have a threonine at the position that is variable between flycatcher species. Likewise, 4 out of 5 members of the Muscicapidae, the family containing the flycatcher genus, have a threonine at the variable position, the collared flycatcher being the sole exception. Ancestral state reconstruction of the variable site suggested that the ancestral state for all passerines was threonine using both parsimony and maximum likelihood.

## Discussion

We report overall conserved gene expression in the caudal telencephalon between 2 flycatcher species. We further find little evidence for transgressive gene expression in F1 hybrids. We first place these results into the broader context of divergence in gene expression in the brain between closely related species and the consequences for hybrids. Second, we outline possible drivers of the species difference in the expression of *RIMS3* and *SYT4* and discuss potential effects of differential expression of these genes on song.

In comparison with the 4 other tissues (testis, heart, liver, and kidney) studied by Mugal *et al*. (2020), the caudal telencephalon has conserved gene expression between the 2 species. The heart, the tissue with the second fewest differentially expressed genes, has nearly 3× the number of differentially expressed genes as the brain region, while testis has nearly 20× (Mugal *et al*. 2020). This result is consistent with Uebbing *et al.* (2016), who reported conserved gene expression between the 2 flycatcher species in the whole brain compared with other tissues. Likewise, previous studies have suggested that species explains a much smaller proportion of variation in gene expression compared with brain region (Drnevich *et al*. 2012; Balakrishnan *et al*. 2014). However, it is unlikely that gene expression in the auditory regions and/or song control system is as conserved as we find in the entire caudal telencephalon, precisely because expression varies across brain regions. The broad, heterogeneous dissected brain sample used in our study is, thus, likely to have diluted the signal of many genes whose expression is limited to small regions or differs in their spatial pattern between species. In line with this, 2 recent studies have reported between 350 and 1,000 differentially expressed genes in individual song nuclei, HVC and RA, across different pairs of estrildid finch species (Wang *et al*. 2019; Shibata *et al*. 2024). Shibata *et al*. (2024), moreover, report comparatively fewer differentially expressed genes in NCM (∼100). This supports not only heterogeneity in expression divergence across brain regions and nuclei, but, in addition, that finer-scale dissections are likely to be more sensitive, since both studies observe substantially more differentially expressed genes between species than the 52 we observe in the flycatcher caudal telencephalon. However, we note that both estrildid species pairs have species divergence times of between 5 and 6 my, more than 5× the age of the flycatcher pair (Wang *et al*. 2019; Olsson and Alström 2020; Davidson and Balakrishnan 2016), and that 913 genes were differentially expressed between the whole brain of zebra finch subspecies with a divergence time of ∼1 my (Olsson and Alström 2020), while between 87 and 197 genes were differentially expressed between the whole brains of the collared and pied flycatchers, which are of similar age (Uebbing *et al*. 2016). This suggests that gene expression may evolve more rapidly in finches compared with flycatchers. Future studies performing RNA sequencing with finer spatial resolution are required to resolve the true level of differential expression in flycatcher auditory and song control regions.

We find differential expression in a small number of genes, 2 of which, *RIMS3* and *SYT4*, are highly expressed in our caudal telencephalon sample. Both *RIMS3* and *SYT4* are members of large gene families encoding proteins involved in neurotransmission and neuroplasticity (Hadley *et al*. 2006; Kiyonaka *et al*. 2007; Kaeser *et al*. 2012; Sudhof 2013). Misexpression of *RIMS3* in humans has been associated with altered cognition (Nishimura *et al*. 2007; Weidenhofer *et al*. 2006, 2009), and *SYT4* is activity-induced, meaning that it can be used as a marker of neural activity (Vician *et al*. 1995), and involved in memory formation (Ferguson *et al*. 2000, 2004; Cunha *et al*. 2009; Dean *et al*. 2009). Both *RIMS3* and *SYT4* are highly expressed in the songbird pallium, the outer region of the telencephalon corresponding to the human neocortex (Reiner *et al*. 2004). *RIMS3* is up-regulated in HVC (proper name), one of the nuclei included in our sampled brain region, relative to other nuclei in the pallium at baseline conditions (Pfenning *et al*. 2014; Whitney *et al*. 2014), but otherwise expressed broadly throughout the telencephalon, both medially and laterally (Lovell *et al*. 2020). As a result, we remain particularly cautious in concluding that the expression divergence between species that we observe in *RIMS3* is due to expression in HVC (proper name), RA, or the auditory regions and not in other parts of the caudal telencephalon that are uninvolved with song perception and production. *SYT4* is up-regulated in HVC relative to surrounding regions (Lovell *et al*. 2020) and is up-regulated in HVC and the auditory lobule when producing (Poopatanapong *et al*. 2006; Wada *et al*. 2006; Whitney *et al*. 2014) and hearing song, respectively (Dong *et al*. 2009). Like *RIMS3*, *SYT4* is expressed throughout the auditory lobule medially, but, in contrast to *RIMS3*, has very low expression levels in the telencephalon, outside of HVC, laterally (Lovell *et al*. 2020). Increased spatial resolution is needed to confirm whether species differences in *SYT4* expression are indeed driven by song-relevant brain regions, such as the auditory lobule and HVC.

Higher expression levels of *SYT4* in the auditory lobule and/or song control system in pied flycatchers compared with collared flycatchers may relate to observed differences between the species in learning plasticity (Bolhuis *et al*. 2000; Bailey *et al*. 2002; Okanoya 2012). Song production in the wild suggests that pied flycatchers commonly learn to produce collared flycatcher song elements where both species co-occur (Haavie *et al*. 2004). In contrast, collared flycatchers are not known to copy song elements from pied flycatchers (Vabishchevich and Formozov 2010). Since both species can learn songs into adulthood, divergent gene expression levels in adult birds have the potential to directly relate to adult song phenotypes. Experimental studies, such as characterizing gene expression following song exposure or singing and/or documenting the behavioral consequences of experimental manipulation of gene expression, would be required to test the role of *SYT4* in song differences between species.

Although we were unable to identify putative regulatory drivers of species differences in expression of *RIMS3*, we found identified species-level divergence in *trans*-acting regulator of *SYT4*. A known upstream regulator of *SYT4*, *STXBP4*, contains a fixed difference between the flycatcher species that may influence its expression. In mammals, the coiled coil motif of *STXBP4* binds to the coiled coil motif of *STX4*, influencing the conformation and binding properties of syntaxin 4-containing complexes (Min *et al*. 1999; Saito *et al*. 2012), while STX4, in turn, regulates *SYT4* (Thomas *et al*. 1999; Harris *et al*. 2016). Although an ortholog of *STX4* has not yet been identified in the flycatcher genome, high-confidence orthologs have been recently identified through transcriptomic approaches in 5 avian species, including a songbird, the zebra finch (Yin *et al*. 2019). Future improvements in the annotations of avian genomes are likely to add additional insight to the drivers of species differences in gene expression.

Even if overall gene expression between species is conserved, the regulatory mechanisms underlying expression levels could vary between species. For example, fixation of alternative *cis*-regulatory variants in 1 species could be compensated by the evolution of corresponding *trans*-regulatory factors, leading to divergence in gene regulation but similar overall gene expression patterns between species. Such compensatory evolution could drive transgressive expression in hybrid individuals if *cis*- and *trans*-regulatory factors from parental species are incompatible. Transgressive expression in the brain is hypothesized to underlie cognitive deficits in hybrid chickadees (e.g. McQuillan *et al*. 2018), suggesting that such changes could have large fitness consequences and may play a role in post-zygotic reproductive isolation. We found transgressive expression in a single gene, *TPH1* (tryptophan hydroxylase 1), necessary for serotonin synthesis in the pineal gland (Patel *et al*. 2004), which, in birds, is located near our sampled brain region. Thus, we consider it likely that greater observed *TPH1* expression levels in hybrids are a byproduct of some samples capturing the pineal gland: expression was confirmed in only 2 out of 10 parental species individuals and 2 out of 3 hybrids, one of which had high expression of N-acetylserotonin O-methyltransferase (*ASMT*), a characteristic pineal gland gene (Supplementary Table 1). Thus, we find limited, at best, evidence for transgressive gene expression in hybrid flycatchers, consistent with previous studies (Davidson and Balakrishnan 2016). Overall, intermediate, rather than transgressive, hybrid gene expression would be predicted to make hybrid songs intermediate to those of the parental species (c.f. Shibata *et al*. 2024). In line with this prediction, the acoustic properties of documented hybrid songs typically lie in between those of the pied and collared flycatcher (Gelter 1987), although this pattern is likely to be under strong influence of social factors.

In conclusion, we report highly conserved gene expression in the caudal telencephalon, containing, among other regions, the auditory lobule and 2 song control system nuclei. It is likely that species differences in gene expression in the auditory lobule and song control system are higher than we estimate for the entire caudal telencephalon. However, we find significant differences between the species in the expression of a singing-related gene, *SYT4*, that is highly expressed in the song control system. Moreover, we identify nonsynonymous fixed differences between the species in a potential upstream transcriptional regulator of *SYT4*. Thus, despite methodological limitations, our analyses reveal divergence in genes regulating neurotransmission and neuroplasticity between 2 closely related songbird species and highlight potential candidate pathways for exploring how and why song learning programs vary across even closely related species.

## Supplementary Material

jkae293_Supplementary_Data

## Data Availability

RNA-seq data are available at https://www.ncbi.nlm.nih.gov/bioproject/ with accession number PRJNA551584. All Supplementary files and code are available in Mendeley Data with DOI: 10.17632/47yjx5z4cp.1 (BioProject; Wheatcroft 2024). Supplementary Table 1 contains TPM (transcripts-per-million reads) for all flycatcher genes and all sequenced tissues. Supplementary Table 2 contains GO (gene ontology) terms for genes expressed in the brain. Supplementary Table 3 contains the results of differential expression analysis for the brain as well as information on species differences and one-to-one human orthologs for each brain-expressed gene. Supplementary Table 4 contains summary information for each module detected by WGCNA. Supplementary Table 5 contains module membership and species associations of each gene expressed in the brain. Supplementary Table 6 lists putative regulators of *RIMS3* and *SYT4*. Supplementary Table 7 contains protein sequences for regulators of *RIMS3* and *SYT4* containing nonsynonymous fixed differences between collared and pied flycatchers. Supplementary Table 8 contains nucleotide sequence information for *STXBP4* for 152 passerine species around the variable site. Supplemental material available at G3 online.
